# Spanish National Registry of Major Osteoporotic Fractures (REFRA) seen at Fracture Liaison Services (FLS): objectives and quality standards

**DOI:** 10.1007/s11657-022-01174-x

**Published:** 2022-11-01

**Authors:** Maria Jose Montoya-Garcia, Cristina Carbonell-Abella, Jose Manuel Cancio-Trujillo, Maria Jesus Moro-Álvarez, Jesus Mora-Fernández, Rafael Izquierdo-Avino, Xavier Nogues, Manuel Mesa-Ramos, Rosa Maria San Segundo-Mozo, Elena Calero-Muñoz, Manuel Naves-Diaz, F. Jesus Olmo-Montes, Enric Duaso, GRUPO REFRA-FLS, Luis del Rio, Jose Luis Fernández-Martín, Daniel Martínez-Laguna, Mª José Miranda, Blanca Hernández, Julia Barrera, Mª Ángeles Vázquez-Gámez, Mercè Giner, Pilar Mesa-Lampre, Leticia Cebollada-Gadea, Manuel Jordan-Jarque, Diana Ovejero Crespo, Maria Jose Robles Raya, Isabel Arnau Barres, Santos Martinez Diaz, Ana María Moreno-Morillo, José Luis Rodríguez-García, Pilar Márquez de Torres, Natalia Angélica Barahona Garibello, Diana Carretero Dios, Antonio José Jiménez Vílchez, Francisco Mesa Ramos, Sara Isabel Copete Marín, María José Justicia Castro, Manuel Valdés Vílchez, Trinidad R. Fernández Ferreras, Urko Díaz Aristizabal, Laura Aguilera Ballester, Jesús Carnicer Cáceres, Esperanza Bienzobas Allue, Laura Sénder, Nuria del Castillo Piñol, Paulina Cuevas Messenger, Mª Dolores Martínez Águila, Carme Ros Bertomeu, Ana Lacal Martínez, Laura Fernández Sénder, Joan Pellejà Carnasa, Christian Alvarado, Andrés Gamboa, Evelyn Irene Alberca, Sofía Alejandra Arriaza, Dolors Grados, Eugenia Sopena, Sonia Castro, Pilar Estudillo, Rami Qaneta, Ramon Fontova, Anna Marsol, Ignacio García Forcada, Gerard Jordà, Indyra Sánchez, Stefan Gálvez, Montse Fibla, Javier Rodríguez

**Affiliations:** 1 Departamento de Medicina, Universidad de Sevilla, Hospital Universitario Virgen Macarena, Sevilla, Spain; 2 Centro de Salud Via Roma ICS Barcelona, Universidad de Barcelona, Barcelona, Spain; 3 Departament of Geriatric Medicine and Palliative Care, Badalona Serveis Assistencials, Badalona Barcelona, Spain; 4 Sección Medicina Interna Hospital Central Cruz Roja San José Y Santa Adela, Madrid, Spain; 5 Universidad Alfonso X El Sabio, Madrid, Spain; 6 Instituto de Investigación Sanitaria del Hospital Clínico San Carlos (IdISSC), Universidad Complutense, Madrid, Spain; 7 Departmento de Traumatologia Y Ortopedia, Hospital Nuestra Señora de Gracia, Zaragoza, Spain; 8 IMIM (Hospital del Mar Medical Research Institute), Centro de Investigación Biomédica en Red de Fragilidad Y Envejecimiento Saludable (CIBERFES), 08003 Barcelona, Spain; 9 Hospital Valle de los Pedroches, Pozoblanco, Córdoba, Spain; 10 Universitat Rovira I Virgili, Tarragona, Spain; 11 Xarxa Sanitària, Social I Docent Santa Tecla, Tarragona, Spain; 12 Hospital Universitario Central de Asturias, Instituto de Investigación Sanitaria del Principado de Asturias, Oviedo, Spain; 13 Unidad de Metabolismo Óseo, Hospital Universitario Virgen Macarena, Sevilla, Spain; 14 Hospital Universitari d’Igualada, FLS Anoia, Igualada, Barcelona, Spain

**Keywords:** Fragility fractures, Osteoporosis, Fracture Liaison Services, Adherence

## Abstract

***Summary*:**

REFRA-FLS is a new registry in Spain aimed at identifying individuals over 50 years of age with a fragility fracture. Using this registry, we found hip fracture is the most prevalent fracture. Treatment for osteoporosis was 87.7%, with 65.3% adherence. REFRA-FLS provides fundamental data in the study of fragility fractures.

**Purpose:**

Fragility fractures are a growing public health concern in modern-aged societies. Fracture Liaison Services (FLS) have been shown to successfully lower rates of secondary fractures. A new registry (REFRA-FLS) has been created to monitor quality indicators of FLS units in Spain and to explore the occurrence and characteristic of fragility fractures identified by these centers.

**Methods:**

We conducted a prospective cohort study based on fragility fractures recorded in the REFRA-FLS registry. Participants were individuals 50 years or above who suffered a low energy fragility fracture identified by the 10 participating FLS units during the study period. The type of FLS unit, the characteristics of the individuals at baseline, along with patient outcomes as quality indicators among those who completed 1 year of follow-up were analyzed.

**Results:**

A total of 2965 patients and 3067 fragility fractures were identified, and the most frequent locations were hip (*n* = 1709, 55.7%) and spine (*n* = 492, 16.0%). A total of 43 refractures (4.5%) and 46 deaths (4.9%) were observed among 948 individuals in the follow-up analyses. Time from fracture to evaluation was less than 3 months in 76.7% of individuals. Osteoporosis treatment was prescribed in 87.7%, and adherence was 65.3% in Morisky–Green test.

**Conclusion:**

Our results provide a comprehensive picture of fragility fractures identified in FLS units from Spain. Overall, quality indicators are satisfactory although a much higher use of DXA would be desirable. As the registry grows with the incorporation of new FLS units and longer follow-up, incoming analyses will provide valuable insight.

**Supplementary Information:**

The online version contains supplementary material available at 10.1007/s11657-022-01174-x.

## Introduction

Clinical relevance of osteoporosis lies in its clinical manifestations, that is, the occurrence of fragility fractures. It has been estimated that, in the western world, roughly 1 in 3 women and 1 in 5 men above 50 years of age will fracture at least once in their remaining lifetime [1]. As populations tend to age, fragility fractures are expected to represent an increasingly serious public health concern. Osteoporosis causes worldwide more than 9 million fractures per year, and accounts for one fragility fracture every 3 s [2]. According to a recent study, 178 million bone fractures occurred worldwide during 2019, most of them occurring in the elderly [3]. Another study estimated that fragility fractures in Europe will increase by 23%, from 2.7 million in 2017 to an estimated 3.3 million in 2030. Associated costs, totaling 37.5 million euros in 2017, are also expected to rise by 27% during this period [4]. The same report estimated a loss of 1 million quality-adjusted life years (QALYs) in 2017 alone due to fragility fractures, and 21 disability-adjusted life years (DALYs) per 1000 individuals aged 50 years and above, exceeding the estimated impact of stroke or chronic obstructive pulmonary disease. Furthermore, all-cause death is increased by more than two times in the year following hip fragility fracture [5].

The risk of fragility fractures varies greatly between territories, with northern European countries experiencing the greatest incidence rates [6]. The ultimate reasons for these disparities remain unknown, but differences in bone mineral density do not seem to play an important role [7, 8]. Within Spain, there are also some differences in the reported incidence rates of fractures between regions. While in Andalusia the incidence rates among individuals over 50 years of age were 123.9 cases and 86.9 cases per 10,000 person years for females and males, respectively [5], the corresponding estimates in Catalonia were 149.7 and 54.6 [9].

The occurrence of a first fragility fracture has been shown to increase the risk of subsequent fractures by 80% [5, 10]. Furthermore, this elevated risk of refracture is concentrated immediately after the initial fracture [5, 11], in what it has been defined as an “imminent risk period.” This period comprises a window of opportunity to identify patients at high risk and to initiate therapies aimed at preventing future fractures.

Despite the available therapeutic arsenal, comprising multiple agents with demonstrated efficacy in reducing the risk of fracture [12, 13], a large treatment gap has been observed among patients with osteoporosis, with a high proportion of untreated eligible patients. The magnitude of this gap was recently estimated between 73 and 63% in European women and men (67% and 60%, respectively, in Spanish population) [4]. A notable treatment gap (up to 72%) has been observed even among women in the first year after a first fragility fracture [14].

Fracture liaison services (FLS) are coordinated models of care for secondary fracture prevention involving multidisciplinary teams of providers. The main goals of FLS are to identify, evaluate, monitor, and treat eligible patients. Thus, patients from a defined geographic area who have experienced a minimal trauma fracture or fragility fracture should be systematically referred to these services to prevent future fractures. This initiative was first introduced in university hospitals in Scotland and gained considerable attention upon demonstrating lower rates of secondary fractures [15]. There is mounting evidence showing that FLS multidisciplinary approach provides a tailored model of care for these patients, with improved relevant patient outcomes such as new fracture rate, treatment coverage, and treatment adherence, along with demonstrated cost-effectiveness [16–18].

Capture the Fracture (CtF) is a global initiative sponsored by the International Osteoporosis Foundation (IOF) aimed to “facilitate the implementation of coordinated, multi-disciplinary models of care for secondary fracture prevention” [19]. CtF has developed a set of international standards and guidelines for best clinical practice that are implemented in a large worldwide network of registered FLS [20]. While the UK has the highest number of FLS on the map for an individual country, Spain, despite the lack of public resources to support this initiative, has the second largest number of FLS. Currently in Spain, all support to FLS is provided by the Spanish Society for Bone and Mineral Research (SEIOMM, according to its initials in Spanish) through the CtF campaign developed by the IOF. The SEIOMM is also behind the FLS-EXCELLENCE and CONSULTING projects, and the creation of the REFRA-FLS registry. The main objectives of these initiatives are (1) to identify reference FLS units that could serve as models for future FLS in Spain, (2) to monitor the occurrence of all types of fragility fractures observed among patients attended in FLS from Spain, and (3) assess the impact of coordinated and protocolized treatment provided at FLS in terms of patient outcomes such as new fracture rate reduction achieved, treatment coverage, or improved adherence.

This report aims to introduce the FLS-EXCELLENCE SEIOMM-FEIOMM project, describing the first results, the initial participating FLS units, and the patients that have been enrolled. We also assess here the current situation of fragility fracture management in Spain by evaluating to what extent participating FLS adhere to the IOF best practice guidelines and quality standards.

## Methods

### Study design and setting

**Fig. 1:**
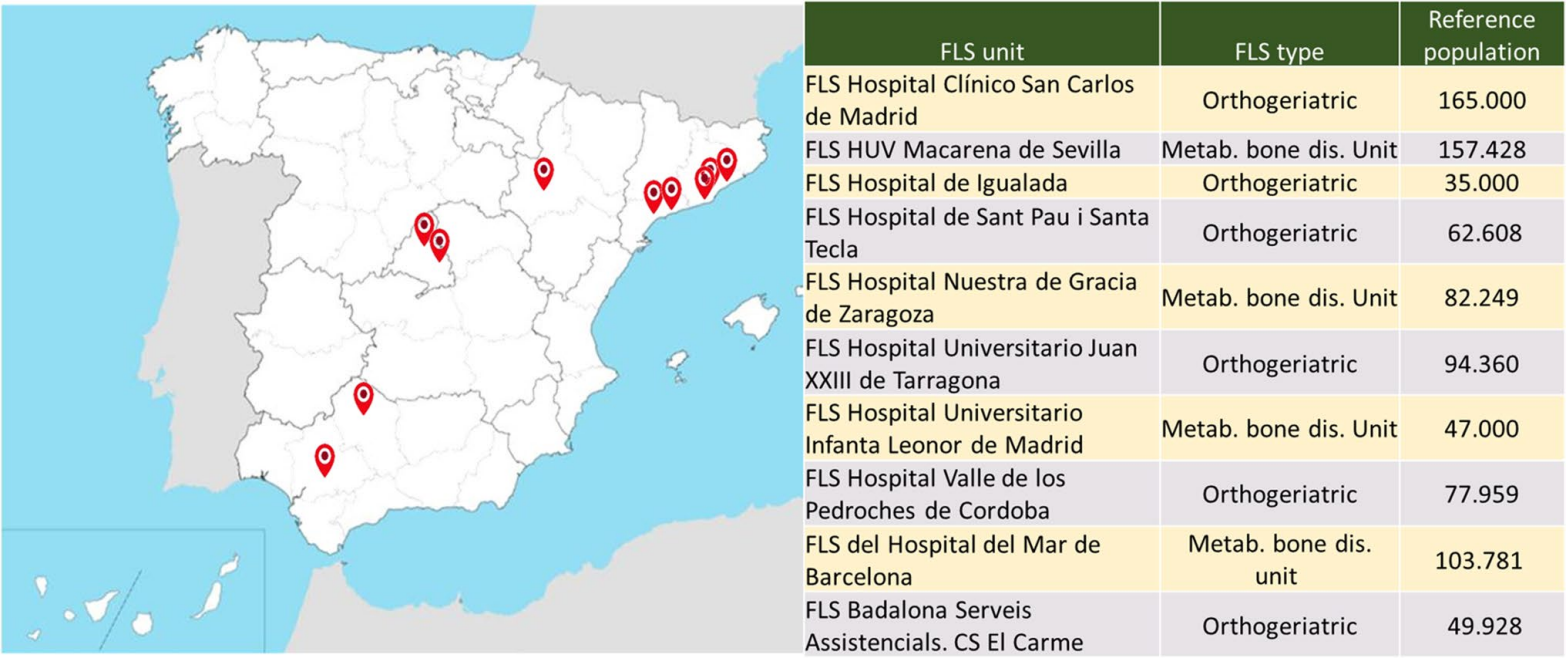
Participating FLS units and catchment area (reference population ≥ 50 years)

FLS-EXCELLENCE SEIOMM-FEIOMM Project aims to prevent secondary fragility fractures and reduce their associated direct and indirect costs by leveraging the creation of FLS units. This project, in line with the CtF global initiative by the IOF [19], has annual calls to incorporate up to six FLS per year to serve as models to foster the creation of new FLS units adapted to different environments. This report describes the results from the first ten FLS units that joined the project during 2018 (*n* = 6) and 2019 (*n* = 4) from throughout Spain (Fig. 1). This is a multicentric, multidisciplinary, prospective cohort study that includes individuals aged 50 years or above that are diagnosed with fragility fracture between June 2018 and September 2021 that are followed with a standardized protocol based on IOF recommendations [19]. Briefly, protocols should include (1) active ascertainment of patients with recent fragility fractures, (2) comprehensive assessment of osteoporotic and refracture risk factors, (3) initiation of interventions based on treatment and/or lifestyle/dietary recommendations, (4) patient adherence monitoring, and (5) systematic data collection.

All FLS units were required to meet the following criteria: (1) active FLS units meeting IOF’s best practice framework excellence criteria with a designated coordinator [21], (2) full coordination between primary care centers from catchment area and hospital-based FLS unit, and (3) comprehensive data collection using SEIOMM’s REFRA-FLS Registry. Participating FLS received a grant from SEIOMM to hire a case manager nurse specifically for this project.

FLS units were classified into two different groups based on the predominant patient profile and the medical department the unit belonged to:

- *FLS-1 (orthogeriatric profile):* Mainly inpatients that are typically identified by the FLS shortly upon first admission with a fragility fracture (mostly hip). The primary goals for these patients are recovery of functional status and to perform a comprehensive future fracture risk assessment. Multidisciplinary teams in these FLS mostly include geriatricians, anesthesiologists, trauma and orthopedic surgeons, and specialists in orthopedic rehabilitation.
- *FLS-2 (metabolic bone disease profile):* Mainly outpatients that are identified by FLS from orthopedic surgery wards, accident & emergency departments, rehabilitation outpatient services, or primary care. Teams in these FLS typically comprise internal medicine specialists, rheumatologists, and endocrinologists.

### Patients

The study population included inpatients and outpatients identified through FLS units, aged 50 years or above that suffered one or more fragility fractures in the prior year. Localization of eligible fractures included hip, dorsal or lumbar spine, proximal humerus, distal radius (wrist), pelvis, and occasionally other localizations recognized as fragility fractures (except for hands, feet, and skull). Exclusion criteria included underlying benign or malignant bone lesions, dementia, advanced cognitive decline, and conditions associated with limited life expectancy. Different proactive strategies were followed by participating FLS to identify new fragility fractures. Thus, periodic meetings with professionals involved in treating patients with fractures (or fracture-related complications) were held to present the FLS unit and the services provided as well as to describe the procedures to refer minimal trauma fracture patients. Furthermore, case manager nurses actively reviewed hospital patient lists (including admissions, visits to emergency departments, and outpatient visits) to identify eligible patients.

### Study procedures and REFRA-FLS registry

**Fig. 2:**
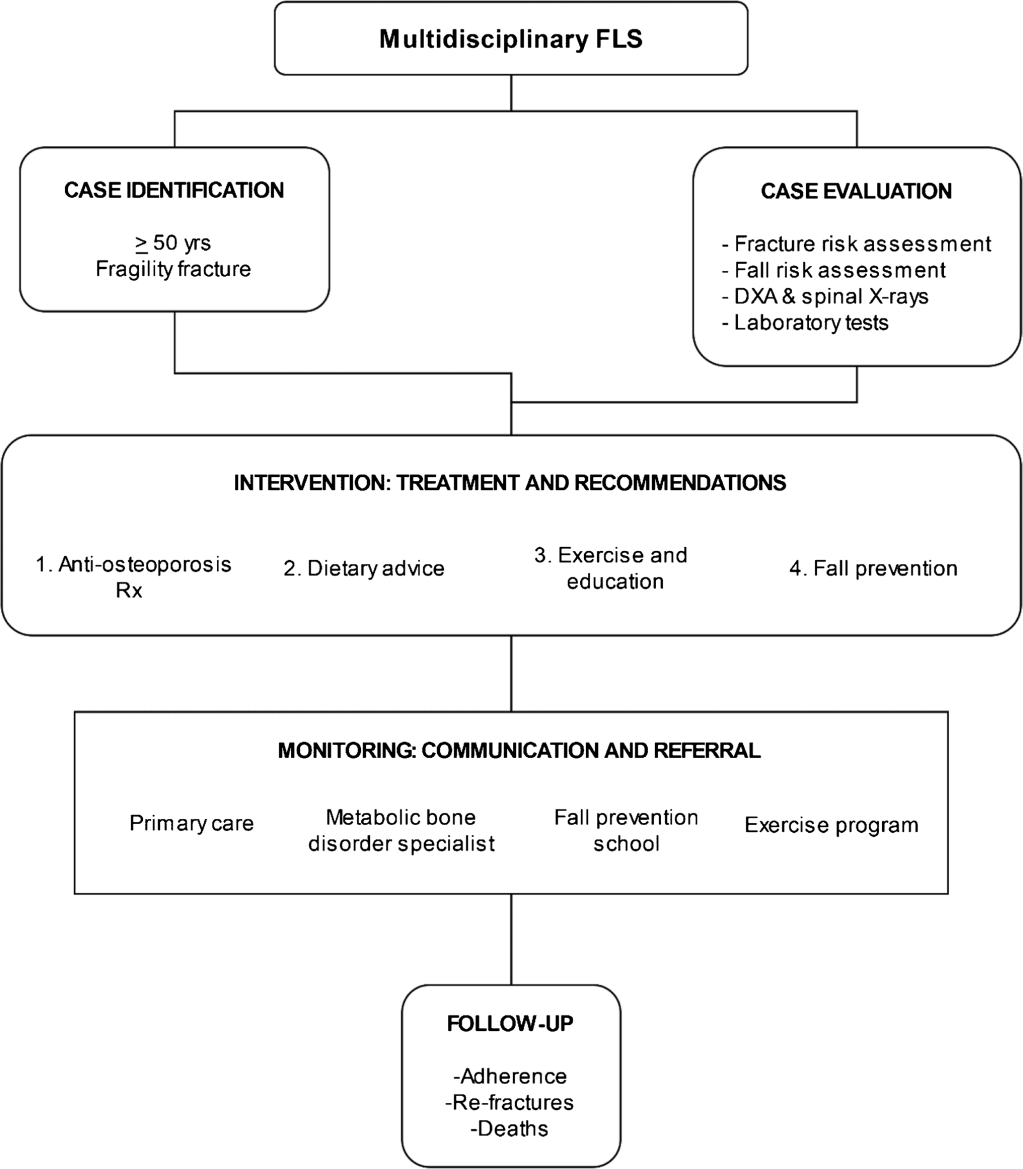
FLS model of care

The study population came from different sources depending on the FLS type as described above. Once eligibility was confirmed by a research nurse, patients were referred to the FLS coordinator (see Fig. 2). After signing an informed consent, comprehensive baseline data is extracted from the patient’s medical records and entered into REFRA-FLS. This questionnaire started with socio-demographic information and details on the index fracture (the one motivating their inclusion in the study). Comorbidity was also recorded including cardiovascular disease (hypertension, dyslipidemia, and ischemic heart disease), gastrointestinal disorders associated with malabsorption syndrome (e.g., celiac disease), endocrine disorders (thyroid/parathyroid disease and diabetes mellitus), and history of neurological (extrapyramidal syndromes), rheumatological (rheumatoid arthritis), respiratory, or renal disease. Relevant medication (anticonvulsant drugs, thyroid medication, aromatase inhibitors, and androgen deprivation therapy) and toxic habits (smoking and alcohol) were also ascertained. Furthermore, the registry includes a fall risk assessment (number of falls in the previous year and “Up and Go” test; low risk was defined as less than 2 falls in the previous year and “Up and Go” test result ≤ 20, and high risk otherwise), a semi-quantitative determination of dietary calcium intake, and 10-year risk of fracture as estimated by FRAX tool [22]. Finally, the baseline assessment included a detailed bone quality assessment with densitometry (with or without TBS), vertebral radiographic morphometry, and biomarkers. Based on all information gathered in this baseline evaluation, participants received tailored recommendations about healthy lifestyle habits and treatments, along with information about the disease in coordination with primary care. Follow-up visits (either in-person or by phone) were performed between 6 and 12 months after the first assessment and annually thereafter. During these visits, adherence to treatment was evaluated by means of a specific questionnaire and by completing the Morisky–Green test [23]. Also in these visits, adherence to healthy habits, and occurrence of side effects, falls, and new fractures after baseline evaluation were ascertained. Finally, reasons for follow-up discontinuation were recorded for each patient.

Calcium daily intake was estimated using SEIOMM’s validated tool [24, 25].

The REFRA-FLS registry uses an encrypted website, protected with username and password, where all activity including logins, visualization, or data modification are recorded. The website can be accessed from computers or tablets with a regular internet connection. This registry complies with all data protection and privacy legislation and standards from Spain and the European Union.

### Analysis

This was a descriptive study. We based our sample size estimation on a reported 3-year cumulative incidence of fracture in the general population of 25% [26]. We hypothesized that this incidence should be reduced by 25% among patients being followed in a FLS. To be able to observe such a reduction with a 90% statistical power, a two-tailed type 1 error of 0.05, and a 15% of lost to follow-up, a minimum of 878 individuals (2634 person-years) should be recruited. The corresponding sample size estimate for a 20% reduction was 1402 (4206 person-years).

Note that follow-up analyses in this report include only those individuals with information available for at least 1 year after entering the study cohort.

Quantitative variables were described using mean and standard deviation (SD) when they followed a normal distribution or median and interquartile range (IQR) otherwise. Categorical variables were described using absolute and relative frequency (percentage). Hypothesis testing for quantitative factors was performed by using Student’s *t* test for those following a normal distribution or Mann–Whitney *U* test otherwise. For categorical variables, chi-square test was used when all cell counts were five or more, and Fisher exact test otherwise. When needed, we performed alternative analyses using logistic regression models to obtain adjusted estimates. All analyses were performed using R for Windows (version 4.0.4).

## Results

### Study population and gender differences

A total of 2965 patients agreed to participate and completed the baseline assessment. Patient recruitment over time is shown in Supplementary Fig. 1. Average daily recruitment during the study period was 2.39 patients. Recruitment was greatly disrupted in FLS-1 units because of the COVID-19 pandemic, going from an average of 1.31 patients per day before March 15, 2020, to 0.55 patients per day thereafter (*p* < 0.001). In contrast, recruitment rate remained virtually unchanged in FLS-2 (1.41 vs. 1.38 patients per day).

Median age at baseline was 82 years (IQR: 73–87). Over 80% of fractures occurred in individuals aged 70 and above. Age at baseline was highest for individuals with hip fracture (85 years, IQR: 79–89) and lowest for those with wrist fracture (71 years, IQR: 64–80). Age was higher among patients from FLS-1 (85 years, IQR: 78–89) compared to those from FLS-2 (78 years, IQR: 70–85; *p* < 0.001). As seen in Table 1, the study cohort included 2396 women (80.8%) and 569 men (19.2%). Since 98 patients (3.3%) presented with two or more initial fractures in different sites, the total number of fragility fractures included was 3067. A total of 1279 fractures came from FLS-1 units whereas 1788 came from FLS-2 units.

**Table 1:** Study population characteristics

Variable	Total (*n* = 2965)	Women (*n* = 2396) (80.8%)	Men (*n* = 569) (19.2%)	*p* value
Age (years) Median (IQR)	82 (73–87)	81 (73–87)	82 (76–87)	0.022
Body mass index (kg/m^2^)				
Mean ± SD	27 ± 11.7	27.1 ± 12.8	26.6 ± 5.6	N.S
Unhealthy habits^a^				
Current smokers *n* (%)	87 (3.9)	50 (2.8)	37 (8.2)	< 0.001
Drinks ≥ 3 alcohol units/day *n* (%)	27 (1.2)	9 (0.5)	18 (4)	< 0.001
Calcium intake (mg/day)^a^				0.040
< 500 mg/day *n* (%)	575 (27.5)	444 (26.5)	131 (31.8)	
500–750 mg/ day *n* (%)	988 (47.3)	791(47,2)	197 (47.8)	
750–1000 mg/day *n* (%)	371 (17.8)	310 (18.5)	61 (14.8)	
> 1000 mg/day *n* (%)	155 (7.4)	132 (7.9)	23 (5.6)	
Index fracture *n*	3067	2488	579	< 0.001
Hip *n* (%)	1709 (55.7)	1285 (51.6)	424 (73.2)	
Spine *n* (%)	492 (16.0)	409 (16.4)	83 (14.3)	
Wrist *n* (%)	342 (11.2)	325 (13.1)	17 (2.9)	
Humerus *n* (%)	265 (8.6)	236 (9.5)	29 (5)	
Other *n* (%)	259 (8.4)	233 (9.4)	26 (4.5)	
Falls (previous year)^a^				
≤ 1 fall *n* (%)	1446 (69.3)	1145 (68.3)	301 (73.2)	0.029
≥ 2 falls *n* (%)	642 (30.7)	532 (31.8)	110 (26.8)	
High risk *n* (%)	1166 (55.8)	924 (55.1)	242 (58.7)	N.S
Ten-year risk of fracture (FRAX)^a^				
≥ 10% major fracture *n* (%)	998 (68.5)	898(76.9)	100 (34.6)	< 0.001
≥ 3% hip fracture *n* (%)	1164 (79.9)	940 (80.5)	224 (77.5)	N.S
BMD using DXA *n* (%)	713 (24.0)	610 (25.5)	103 (18.1)	< 0.001
Osteoporosis *n* (%)^a^	401 (56.2)	342 (56.1)	59 (57.3)	
Osteopenia *n* (%)^a^	269 (37.7)	232 (38)	37 (35.9)	
Normal *n* (%)^a^	43 (6)	36 (5.9)	7 (6.8)	
Prior fractures *n* (%)	966 (32.6)	867 (36.2)	99 (17.5)	< 0.001
Prior osteoporosis Dx *n* (%)	665 (31.8)	591 (35.2)	74 (18.0)	< 0.001
Prior osteoporosis Rx *n* (%)	450 (15.2%)	428 (17.9)	22 (3.9)	< 0.001
Comorbidities				
Cardiovascular disease *n* (%)	1446 (65.2)	1154 (65.2)	292 (65)	
Gastrointestinal disease *n* (%)	115 (5.2)	90 (5.1)	25 (5.6)	
Endocrine disorders *n* (%)	633 (28.5)	522 (29.5)	111 (24.7)	
Neurological disease *n* (%)	392 (17.7)	288 (16.3)	104 (23.2)	0.001
Respiratory disease *n* (%)	261 (11.8)	159 (9)	102 (22.7)	0.001
Urolithiasis *n* (%)	14 (0.6)	10 (0.6)	4 (0.9)	
Chronic kidney disease *n* (%)	194 (8.7)	132 (7.5)	62 (13.8)	< 0.001
Rheumatoid arthritis *n* (%)	33 (1.5)	31 (1.8)	2 (0.4)	
Comedication	305 (14.6)	261 (15.6%)	44 (10.7%)	0.0015
Thyroid medication n (%)	165 (53.6)	156 (59.3)	9 (20)	< 0.001
Corticosteroids *n* (%)	106 (34.4)	81 (30.8)	25 (55.6)	0.002
Anticonvulsant drugs *n* (%)	42 (13.6)	33 (12.5)	9 (20.0)	
Aromatase inhibitors *n* (%)	23 (7.5)	23 (8.7)	0 (0.0)	
Androgen deprivation *n* (%)	8 (2.6)	3 (1.1)	5 (11.1)	0.001

Data presented as the number of patients (percentage). Osteoporosis BMD, T-score less than − 2.5; osteopenia BMD, T-score greater than − 2.5 to less than − 1; normal BMD, T-score greater than − 1

*SD* standard deviation, *IQR* interquartile range, *N.S.* not statistically significant

^a^Among patients with data available

The distribution of study participants by gender and fracture site among other factors is shown in Table 1.

The most frequent fracture site was hip (55.7%, *n* = 1709), being more prevalent among men (73.2%, *n* = 424) than among women (51.6%, *n* = 1285; *p* = 0.001). Furthermore, fracture site varied greatly depending on the type of FLS unit (*p* = 0.001). Thus, in FLS-1, the most common site was hip (75.3%), followed by spine (7.8%), humerus (2.7%), and wrist (1.4%). In contrast, in FLS-2 hip fractures accounted for only 41.8%, followed by spine (21.9%), wrist (18.0%), and humerus (12.8%).

Median time from fracture to FLS admission was 13 days (IQR: 2–87.2), being longer among females (median = 15, IQR: 2–91) than among males (median = 8, IQR: 1–64.5; *p* = 0.0001). Time to FLS admission was also shorter for pelvis (median = 2, IQR: 1–11.8) and hip fracture (median = 4, IQR: 1–18), and longest for wrist (median = 88, IQR: 43.2–116.8). Finally, time to FLS admission was only 2 days (IQR: 1–4) in FLS-1 compared to more than 2 months (71 days; IQR: 15.2–117) in FLS-2 (*p* = 0.0001).

Calcium intake was between 500 and 1000 mg/day for most individuals but was found to be under 500 mg/day in 27.5%, with no sex differences noted (Table 1).

The risk of falling was high in 55.8% of study patients and unrelated to sex. Only 50.8% made no use of walking aids, 24.5% used a walking stick, 21.1% required either two walking sticks or a walker, and 3.9% required a wheelchair.

At baseline, 68.5% of participants had over 10% risk of a major fracture in the following 10 years, as measured by FRAX tool. This estimate differed between sexes (*p* = 0.001) and location of index fracture (*p* = 0.0001). Thus, while only 34.6% of men were at high risk of fracture (i.e., over 10%) according to FRAX, this percentage was 76.9% for women. Likewise, the proportion of individuals at high risk of major fracture ranged from 9.8% among patients with wrist index fracture and 16% among those with hip index fracture. Furthermore, 10-year risk of hip fracture ranged from 3.6% among patients with a wrist index fracture to 7.6% among those with hip index fracture (p < 0.001). When adjusted by age, this difference disappeared (*p* = 0.940).

Bone mineral density was evaluated using DXA in spine and/or hip in 713 participants (24.0%). This assessment was performed more frequently among women (25.5%) than among men (18.1%; *p* = 0.001) and more frequently among patients up to 65 years of age (47.3%) than among those above 65 years (21.1%; *p* < 0.001). These proportions also differed by index fracture site: 39.8% (spine), 38.0% (wrist), 31.7% (humerus), 16.0% (hip), and 21.2% (other sites). Patients with recorded densitometry were classified according to WHO criteria into osteoporosis, osteopenia, or normal BMD as seen in Table 1.

A previous fragility fracture (i.e., before the index fracture) or a previous osteoporosis diagnosis was more frequent among women than men (Table 1; *p* = 0.001). Overall, osteoporosis treatment at baseline was scarce, being lower among males (*p* < 0.001). Even among those with an osteoporosis diagnosis, only 34.4% received treatment, again being less frequent among men than women (10.8% vs. 37.4%; *p* < 0.001) The most frequent drugs being used were bisphosphonates (alendronate, 41.6%; risedronate, 12.1%; ibandronate, 8.2%), followed by denosumab (23.7%), teriparatide (7.6%), and raloxifene (1.0%).

Table 1 shows the frequency of comorbidity as well as relevant concomitant medication use among study patients.

### Study outcomes

**Fig. 3:**
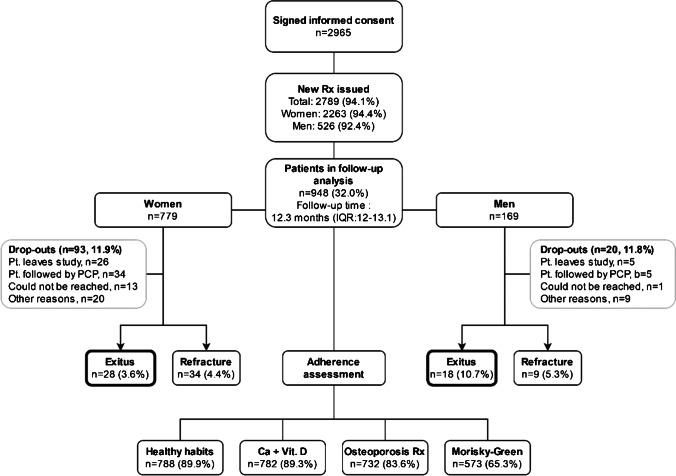
Follow-up chart

After a comprehensive clinical evaluation, FLS staff provided recommendations on healthy lifestyle habits and prescribed new pharmacological treatments for 2927 patients (94.1%; Fig. 3). This percentage did not significantly differ between sexes (94.4% and 92.4% in females and males, respectively). Among these treated patients, 54.9% received calcium supplements, 94.3% received vitamin D supplements, and 87.7% received a specific osteoporosis treatment. This specific treatment was mainly (92.1%) antiresorptive therapy (oral bisphosphonates, 40.8%; denosumab, 36.9%; IV bisphosphonates, 14.4%), while some patients received bone-forming therapy with teriparatide (7.9%). Sex did not seem to influence the pattern of drug prescriptions except for teriparatide (females: 9.2% vs. males: 2.9%; *p* = 0.0001). We found notable differences in prescribed treatments according to the FLS types. While vitamin D supplements were widely prescribed in both (95.3% and 94.6% in FLS-1 and FLS-2, respectively), calcium supplements were significantly more frequent among patients from FLS-1 (76.4%) than FLS-2 (40.6%; *p* < 0.001). The most frequent osteoporosis treatment in FLS-1 was denosumab (49%) followed by zoledronic acid (25.4%), alendronate (18.8%), teriparatide (6.2%), and risedronate (0.5%). In contrast, the most prescribed agents in FLS-2 were alendronate (46.3%), followed by denosumab (29.2%), teriparatide (8.9%), risedronate (7.6%), and zoledronic acid (7.5%).

**Fig. 4:**
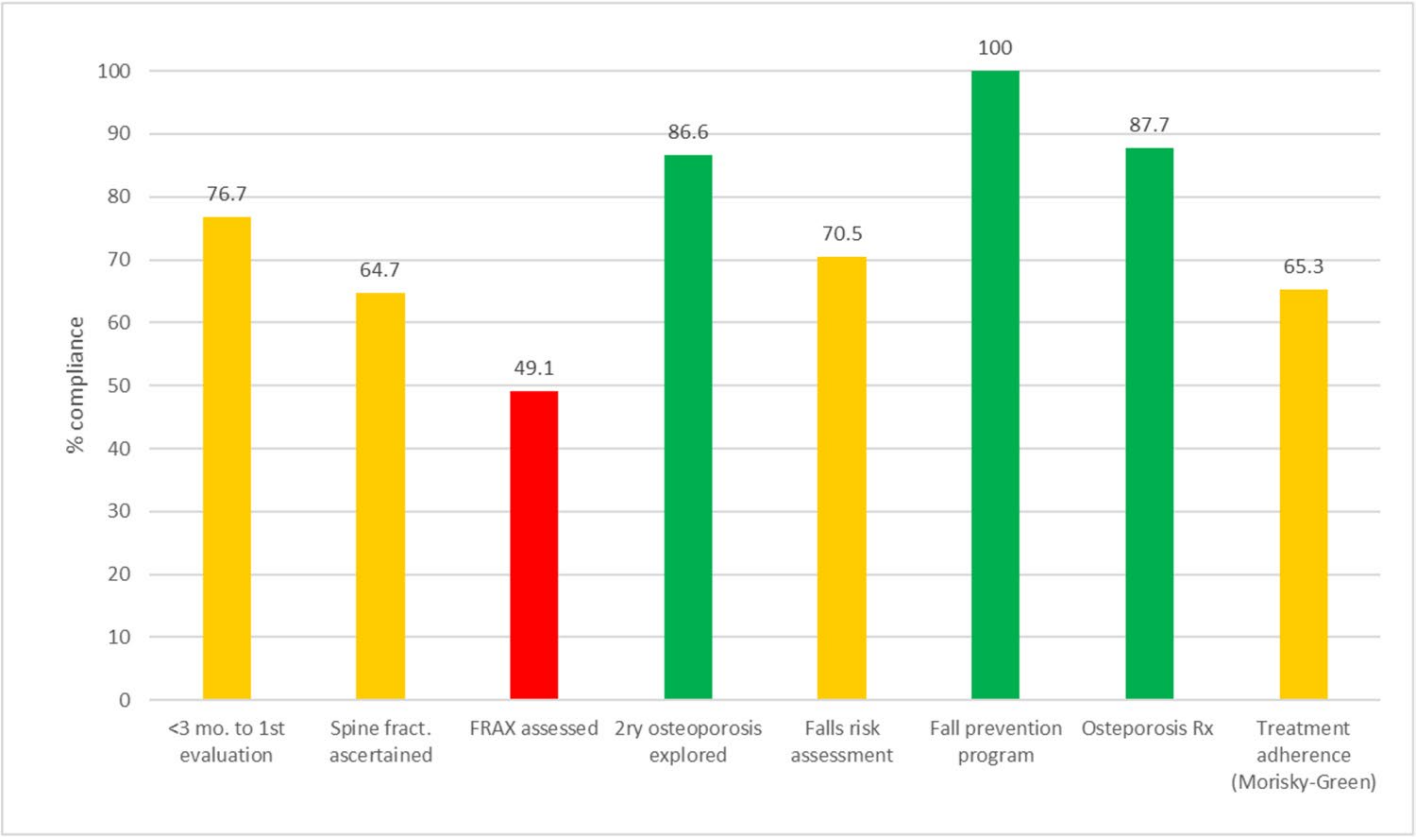
Compliance with quality standards. Colors in bars indicate levels of achievement: red, 0–49%; amber, 50–79%; green, 80% or higher

Throughout the study period, FLS coordinators from all participating FLS units held quarterly meetings with SEIOMM’s representatives to update assessment and treatment recommendations aligned with the European guidance for the diagnosis and management of osteoporosis in postmenopausal women [27] and the American Association of Clinical Endocrinologists (AACE) [28]. Figure 4 describes to what extent different key performance indicators were met by the FLS participating units. We found that time to first evaluation was less than 3 months after the index fracture in 2351 patients (76.7%) and less than 6 months in 2837 (92.6%). Radiographic assessment of vertebral fractures was performed in 1918 patients (64.7%), with similar proportions in males and females. Ten-year risk of fracture based on FRAX tool was estimated in 49.1% of patients. Laboratory tests, aimed at exploring secondary causes of osteoporosis, were performed in 86.6% of patients, while fall risk was evaluated in 70.5%. Fall prevention units were only available for 45.8% patients with an incident fracture, with 12.7% of patients being referred to these units. However, fall prevention programs (that included recommendations on specific exercises to be completed) were initiated in all patients by research nurses. Prevalence of unhealthy habits was assessed in 74.8% of patients, while walking ability and consumption of dairy products were ascertained in 70.0% and 70.5% of patients, respectively.

Communication between FLS and primary care physicians was mostly through clinical reports, or directly via email or telephone calls. Occasionally, other communication strategies such as meetings to present FLS units and their procedures were used. With rare exceptions, no active search of patients with fragility fractures was performed in primary care. Therefore, proactive identification of minimal trauma fracture was mostly limited to patients attending specialized care.

To date, a total of 948 patients (779 women and 169 men) have been followed for at least 1 year, with a median follow-up of 12.3 months (IQR: 12.0–13.1; Fig. 3). Follow-up was mostly conducted via telephone (86.5%). During this period, a total of 46 patients (4.9%) have died, with mortality being significantly higher among men (*p* < 0.001), and 43 (4.5%) patients have experienced a new fracture. Self-reported compliance to healthy habits recommendations, calcium and vitamin D supplements, or prescribed osteoporosis treatment was over 80%. According to the results of the Morisky–Green tests, adherence to disease specific treatment was somewhat lower, 64.3% among females and 70.3% among males. When FLS type was considered, we found that therapeutic adherence as estimated by these tests was higher in FLS-1 (75.2%) than in FLS-2 (58.0%; *p* < 0.001). Notably, small differences in mortality were observed between FLS types, slightly higher in FLS-1 (6.7%) than in FLS-2 (3.4%; *p* = 0.027) but not in occurrence of re-fracture events (*n* = 19, 4.6% and *n* = 24, 4.5%, respectively, as seen in Fig. 3).

## Discussion

We were able to identify 2965 patients with a recent osteoporotic fracture attending FLS units in Spain. The geographical distribution of these units makes the study sample quite representative of the Spanish general population. Although previous publications described individual Spanish FLS units [29–31], this is the first multicenter study aimed at combining data from multiple FLS units within Spain. The fact that a homogeneous protocol regarding identification, evaluation, and treatment initiation was followed in all Units and the quarterly monitoring by SEIOMM group of experts makes this study unique and strengthens clinical results relevance.

The pace in patient recruitment significantly slowed down after the outbreak of COVID-19 pandemic. This could be related to different intertwined factors such as the deprioritization of non-COVID medical care, patients’ reluctance to attend health care centers for non-urgent care, high mortality among elderly patients who are most susceptible to fractures, or lower incidence rates of fractures. The impact of the pandemic was rather different depending on the type of FLS unit, with FLS-1 units being the ones primarily affected. Since hip fractures comprise two-thirds of all fractures from these units, and considering that these fractures typically require hospitalization, it seems plausible that some incidence rate decrease (at least of hip fracture) did occur during the study period.

We found that the patient profile differed greatly in the two types of FLS units, previously described as FLS-1 and FLS-2. Although both provided multidisciplinary and coordinated care, one was focused on new admissions of patients presenting mainly with hip fracture, while the other included mostly younger outpatients with early osteoporotic fracture at various sites. These differences are inherent to the distinct origins of this units, and explain observed differences in some study outcomes, such as the longer time to evaluation in FLS-2 (due to patients being captured in routine outpatient visits rather than upon fracture-related hospital acute admissions) or the greater mortality in FLS-1 (that tends to capture older patients). Although currently both share the same FLS systematic methodology, patient profile remains conditioned by their background and the medical specialties of the clinicians in charge of these units (either orthogeriatrics or bone metabolism). Thus, although these differences in patient profiles are unlikely to disappear in the near future, acknowledging them contributes toward improving quality of care in both FLS types. Overall, we observed a shortage of vertebral fractures irrespective of FLS type. Since many of these fractures do not result in a hospital admission, greater coordination with primary care and radiology departments would be needed to fully capture them.

Patient characteristics were similar to previous studies showing a low percentage of osteoporosis diagnosis (despite high prevalence of earlier fragility fractures), high 10-year risk of fractures, high prevalence of gait disorders, high risk of falls, low calcium intake, and limited use of specific osteoporosis treatment. Furthermore, we were able to replicate the gender gap in treatment and diagnosis reported by other authors [32–34]. Along these lines, the high proportion of hip fractures among men (or the low proportion of other fragility fractures) suggests that osteoporosis burden among men remains largely overlooked.

A set of key performance indicators have been proposed to measure the effectiveness of FLS and assist in quality improvement [20]. Our results were rather satisfactory, with some indicators showing quite a high level of achievement such as the high proportion of patients undergoing comprehensive assessments within 3 months, or the percentage of patients initiating and maintaining osteoporosis treatment after 1 year of follow-up. More modest results, yet still satisfactory, were observed for identification of spine fractures (64.7%) and falls risk assessment (70.5%).

On a more negative note, we observed that DXA was performed only in 1 out of 4 patients in the study population. This finding could be related to the drastic drop in non-essential procedures during the COVID pandemic and to high proportion of hip fractures and elderly patients in the study population. In fact, around 50% of all patients up to 65 years of age underwent DXA. However, considering the valuable information that DXA provides for evaluating the risk of fracture risk and therapeutic goals [35, 36], a more widespread use of this technique would be desirable.

Regarding osteoporosis treatment, we also found that initiation of bone-forming agents in our study population was low, despite both national [37] and international guidelines [27, 28] recommending sequential treatment with a bone-forming agent followed by antiresorptive therapy in patients at very high risk of fractures. Finally, we found that communication between FLS units and primary care was not as fluid as it should be. Full coordination with primary care is essential to ensure the continuity of care which would improve secondary prevention of fragility fractures [38], and this should be considered to improve FLS units.

It is widely accepted that risk of refracture in the first months after a first event is particularly high [39]. We have shown moderate refracture rates among the study population after completing 1 year of follow-up in FLS units. Implementing the FLS methodology also resulted in bridging the reported sex gap in terms of evaluation, diagnosis, and treatment of this condition.

One of the achievements of this project was the creation of a national registry of fragility fractures in Spain. This has been key to assessing the quality of care in the different FLS, enabling comparisons between individual FLS within the registry. Furthermore, as national registries grow in number and size with time, they will allow to perform interesting analyses with comparisons between countries. These initiatives are also crucial to monitor the effectiveness of the existing model of care, promoting its continuous improvement, and to increase awareness among policymakers and health professionals [40, 41]. Collaborative efforts aimed at sharing experiences of existing national registries and providing guidance to upcoming registries such as the “The Hip Fracture Registry Toolbox” are much appreciated [42].

Our study has some limitations. Information on the expected number of non-vertebral and hip fractures was not available from the ten specific geographical areas where these FLS are located and therefore we could not estimate the proportion of expected fractures that were captured. Patient follow-up is relatively short in this first analysis, which prevents drawing conclusions about the efficacy of this model of care in terms of adherence, prevention of future fractures, or cost-effectiveness. However, the fact that this is the first multicenter registry of major osteoporotic fractures at a national level is of great value since it provides information on the characteristics of the patients and the fractures that lead them to consult, in addition to highlighting areas for improvement in each of the units. Furthermore, the project, backed by the SEIOMM, is being expanded to include new FLS from some Spanish areas that are currently underrepresented. This will boost the statistical power and provide an even broader picture of the state of the disease in Spain and the contribution of FLS. On the other hand, extrapolating our results to those from other populations might not always be possible. For instance, it has been shown that while FRAX is a useful tool to estimate future risk of hip fracture, it seems to underestimate the risk of major osteoporosis fracture in the Spanish postmenopausal women [43].

The results of this study highlight the benefits of establishing a nationwide computerized registry such as like *REFRA-FLS* to monitor the performance of different types of FLS. While overall quality indicators are quite satisfactory, we were able to identify some areas for improvement, such as the low use of DXA or the observed delay in capturing some fractures, especially those that do not involve hospital admission. Integrating orthopedic surgeons and rehabilitators in FLS multidisciplinary teams (especially in FLS-2, with metabolic bone disease profile) could help identify and assess patients with osteoporotic fractures earlier. Overall, we found this initiative not only helps improve current FLS units by monitoring their performance but could serve to promote the creation of new FLS, which has been shown to be the most successful strategy to fight osteoporotic fractures and their associated morbidity and mortality.

## Supplementary Information

Below is the link to the electronic supplementary material.

Supplementary file1 (DOCX 189 KB)

## Data Availability

Please contact author for data requests.
