# Supplementary figures and images for: Spanish National Registry of Major Osteoporotic Fractures (REFRA) seen at Fracture Liaison Services (FLS): objectives and quality standards
Source: Arch Osteoporos. 2022 Nov 1;17(1):138. doi: 10.1007/s11657-022-01174-x (PMC9626427; doi:10.1007/s11657-022-01174-x)

**Supplementary Figure 1. Patient recruitment over time.**


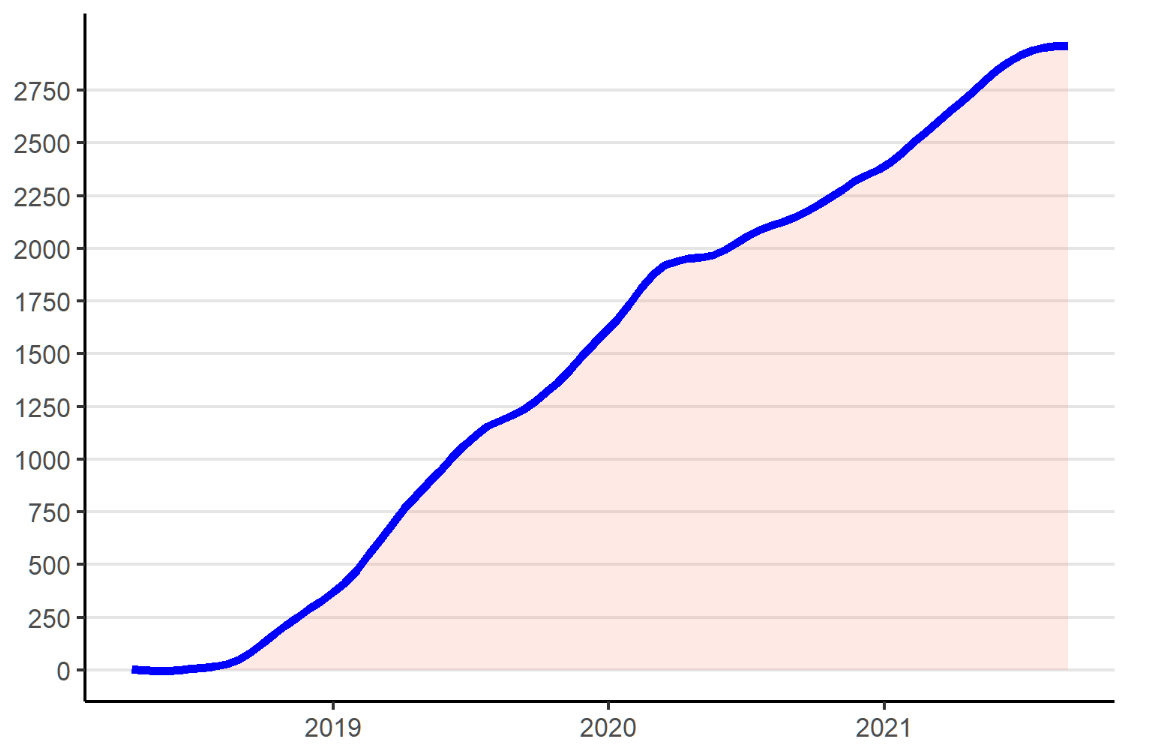

Supplement: Supplementary file 1 — Supplementary file1 (DOCX 189 KB) [file 11657_2022_1174_MOESM1_ESM.docx]
